# Semiautomatic Epicardial Fat Segmentation Based on Fuzzy c-Means Clustering and Geometric Ellipse Fitting

**DOI:** 10.1155/2017/5817970

**Published:** 2017-09-20

**Authors:** Vladimir Zlokolica, Lidija Krstanović, Lazar Velicki, Branislav Popović, Marko Janev, Ratko Obradović, Nebojsa M. Ralević, Ljubomir Jovanov, Danilo Babin

**Affiliations:** ^1^Department of Fundamentals Sciences, Faculty of Technical Sciences, University of Novi Sad, Trg Dositeja Obradovića 6, 21000 Novi Sad, Serbia; ^2^Faculty of Medicine, University of Novi Sad, Hajduk Veljkova 3, 21000 Novi Sad, Serbia; ^3^Department of Power, Electronic and Telecommunication Engineering, Faculty of Technical Sciences, University of Novi Sad, Trg Dositeja Obradovića 6, 21000 Novi Sad, Serbia; ^4^Mathematical Institute, Serbian Academy of Arts and Sciences, Kneza Mihaila 36, 11000 Beograd, Serbia; ^5^Faculty of Engineering and Architecture, imec-IPI-UGent, Ghent University, Sint-Pietersnieuwstraat 41, 9000 Gent, Belgium

## Abstract

Automatic segmentation of particular heart parts plays an important role in recognition tasks, which is utilized for diagnosis and treatment. One particularly important application is segmentation of epicardial fat (surrounds the heart), which is shown by various studies to indicate risk level for developing various cardiovascular diseases as well as to predict progression of certain diseases. Quantification of epicardial fat from CT images requires advance image segmentation methods. The problem of the state-of-the-art methods for epicardial fat segmentation is their high dependency on user interaction, resulting in low reproducibility of studies and time-consuming analysis. We propose in this paper a novel semiautomatic approach for segmentation and quantification of epicardial fat from 3D CT images. Our method is a semisupervised slice-by-slice segmentation approach based on local adaptive morphology and fuzzy c-means clustering. Additionally, we use a geometric ellipse prior to filter out undesired parts of the target cluster. The validation of the proposed methodology shows good correspondence between the segmentation results and the manual segmentation performed by physicians.

## 1. Introduction

Epicardial fat presents a deposition of a visceral fat that surrounds the heart and is mainly located in the atrioventricular and interventricular groves surrounding the epicardial vessels [1]. There are studies that suggest that growing amount of epicardial fat can indicate the development of atherosclerotic diseases and correlate well with the parameters of the metabolic syndrome. Further on, some studies suggest that increased epicardial fat volume and its outspread distribution play an important part in the development of an unfavorable metabolic profile and potentiate cardiovascular risk [2]. Some of the conditions linked to increased epicardial fat volume include the following: increased prevalence of coronary artery calcifications [3, 4], impaired diastolic filling [5], development of myocardial infarction [3] and atrial fibrillation [6], and increased carotid stiffness [7]. Finally, there is evidence that epicardial fat possesses characteristics of an endocrine tissue capable of promoting accelerated atherosclerosis [8, 9].

Due to the importance of the epicardial fat and its relation to various cardiovascular and metabolic diseases, epicardial fat detection, quantification, and 3D segmentation have become important topic in cardiac image analysis and cardiology in general. Different imaging technologies can be used for epicardial fat imaging, segmentation, and quantification [10–12]. Specifically, CT has been found to be more accurate in the evaluation of fat tissues due to its higher spatial resolution when compared to ultrasound [13].

In Coppini et al. [13], a region growing strategy is proposed as preprocessing step, in order to extract the heart region from the rest of the target CT image. As an additional step, medical doctor was required to place up to 7 control points along the pericardium border, which are then used for spline interpolation in order to obtain a smooth closed pericardial contour. Finally, the epicardial fat is simply quantified by thresholding, since it is theoretically located within this generated contour. A hierarchical, multiclass, multifeature, fuzzy affinity-based computational framework for segmentation of abdominal adipose tissue was proposed in [14] and further extended in [15] in order to be applicable to segmentation of epicardial fat. Further on, the work of [13] additionally reduced the user intervention, although the expert was still required to scroll through slices and to place control points on the pericardium. An algorithm that uses the anatomy of the heart to detect the pericardium line and separate epicardial fat from pericardial fat is presented in [16]. In Dey et al. [17], the authors used an additional processing step for the identification of the pericardium by tracing lines going from the heart centroid to the pericardium layer and using splines to interpolate outer points. Shahzad et al. [1] proposed the first fully automated method for epicardial fat segmentation that is using multiatlas approach. In Zlokolica et al. [18], segmentation of the heart region from the rest of the image content was done by applying morphological opening by reconstruction operation followed by thresholding and subsequent morphological dilation operator. The size and the shape of the morphological operators are found experimentally for all image slices, while the threshold value is found by statistical analysis of the input image histogram and is in general different and adaptable for each image slice. That idea is further extended in [19] by imposing structural prior knowledge and subjective-objective correspondence, as an additional step for excluding the segmentation parts that were out of the predefined circle.

In this paper, we build on our previous work and propose a new method for epicardial fat segmentation which is based on a 2D segmentation approach that includes fuzzy c-means clustering and ellipse fitting. The fuzzy c-means clustering is done using a predefined number of clusters within a detected region of interest (ROI). The clustering is performed with reduced supervision, where the only input information from a physician are the coordinates of a single patch corresponding to the epicardial fat tissue. After the user marks a patch corresponding to the epicardial fat, the proposed fuzzy c-means clustering is applied to segment the correct target image cluster as the one with the minimal distance to the user-marked patch. We use the following facts as prior information in our method. Firstly, the epicardial fat tissue lies in the neighborhood of the ellipse placed at the center of the mass of the resulting cluster. This allows us to determine the epicardial fat pixels in segmentation process. Secondly, motivated by the fact that the epicardial fat is located on the surface of the ventricles (mainly anterolateral surface), we impose additional assumptions regarding the angle in the parametrization of the ellipse. By doing so, we additionally filter parts of the resulting cluster not belonging to the epicardial fat tissue. We validate the proposed concept by comparing the resulting segmentation results to the manual segmentation provided by a cardiovascular surgeon. Additionally, we also demonstrate that the proposed approach outperforms the segmentation method of [18] and general image segmentation methods: snake active contour method [20] and region growing method [21].

The paper is organized as follows. In Section 2, we describe the proposed methodology for epicardial fat segmentation, where we first discuss input data set and introduce image preprocessing step in Section 2.2. After that, we explain the proposed segmentation approach in Section 2.3. Experimental results with discussion are provided in Section 3. Finally, the conclusions are drawn in Section 4.

## 2. Materials and Methods

In this section, we discuss data set used in our study and propose methodology for epicardial fat segmentation. The proposed method consists of two main steps: (i) image preprocessing and ROI extraction (Section 2.2) and (ii) our novel image segmentation approach (Section 2.3) used for final epicardial fat segmentation. Section 2.3 is organized in two subsections: (i) Section 2.3.1 explaining the proposed fuzzy c-means clustering step and (ii) Section 2.3.2 describing the cluster data filtering step by geometrical fitting scheme.

### 2.1. Data Set and Ethical Approval

The study included a total of ten patients with previously established coronary artery disease (stable and nonstable angina). All patients were submitted to standard contrast chest CT scan including CT coronarography performed on a Siemens SOMATOM Dual Source 256-slice scanner. In the proposed approach, we consider contrasted CT images with 12 bits pixel depth resolution, providing thus 4096 gray levels. The study was approved by the institutional review board, and all subjects signed the written informed consent. The study was conducted in accordance with the Declaration of Helsinki.

### 2.2. CT Image Enhancement and ROI Extraction

**Figure 1:**
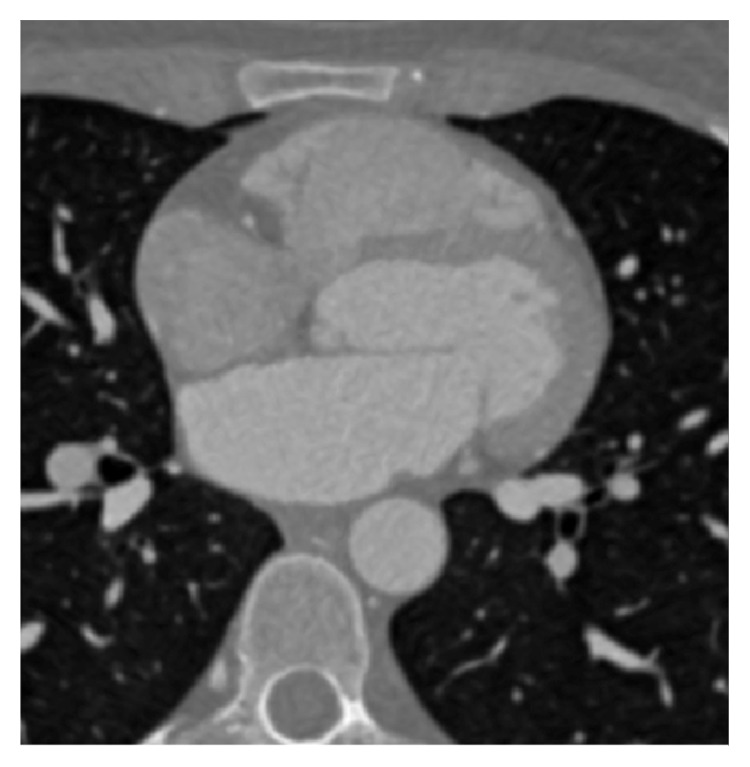
Input CT image slice of a heart, without additional contrast enhancement applied.

In this section, we build on our previous work [18] for enhancing input CT image slices and detection of heart (and epicardial fat) region of interest (ROI). The goal of the processing step is to remove the nonheart-related image content and retain image content only related to the whole heart region. An example of one slice of acquired 3D CT image of a heart, without previous contrast enhancement, is shown in Figure 1.

As can be seen from Figure 1, the input image slice is not of sufficient image quality to be used for manual or automatic image segmentation. In order to improve input image slice quality in terms of visual quality and the quality for feature extraction and segmentation, we propose to first preprocess the input image slices with image-adaptive tone mapping. The proposed tone mapping function is formed based on input image histogram based on which the transfer tone mapping function is optimized in terms of its parameters.

**Figure 2:**
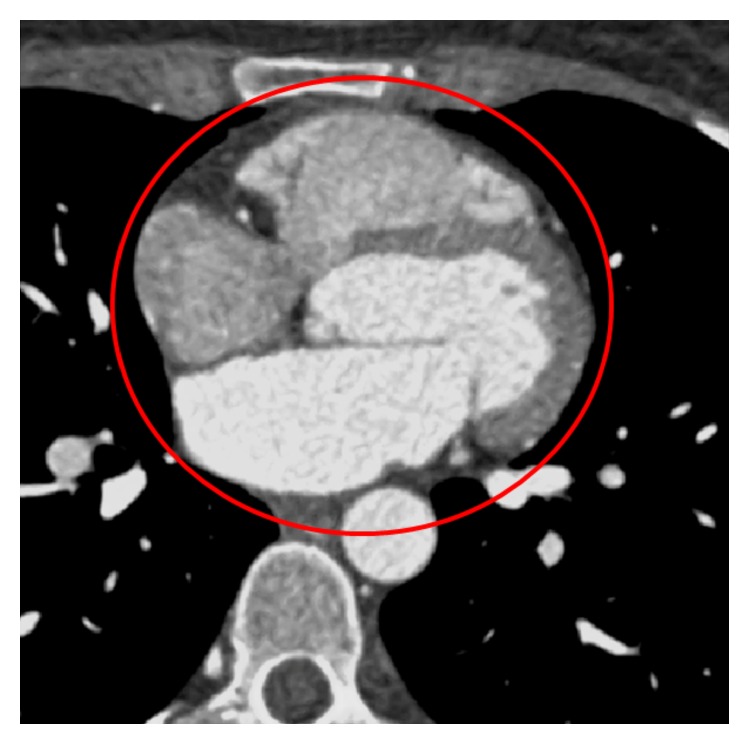
Output image after contrast enhancement with denotation of the region of interest that corresponds to the heart.

We aim at distinguishing between the whole heart region and surrounding (other) parts of the body. For this reason, we first detect ROI that includes the whole heart (denoted in Figure 2) within which we further perform 3D segmentation of epicardial fat. Prior to this, we apply contrast enhancement on input image to facilitate subsequent segmentation task.

**Figure 3:**
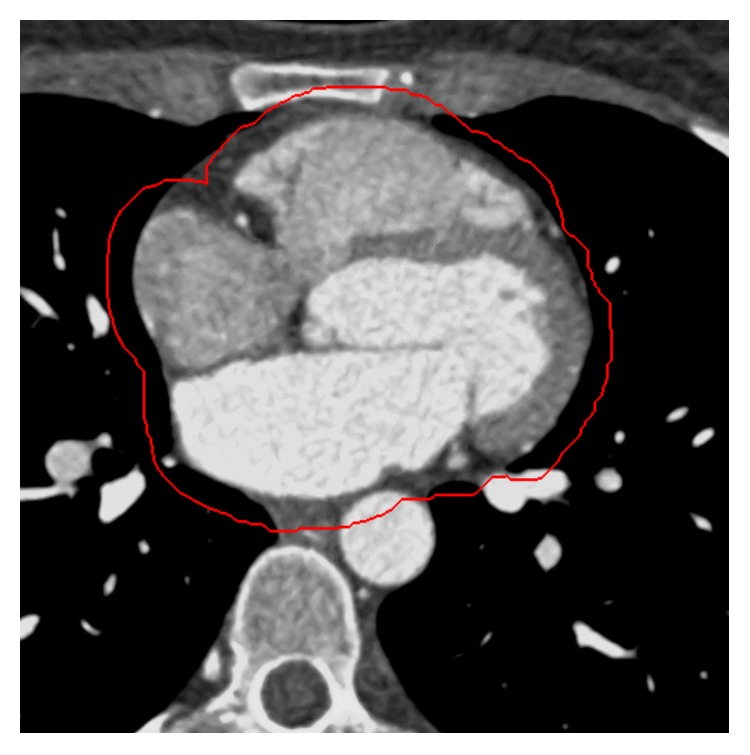
The result of automatic ROI extraction from the rest of the image content (rounded by red line), for a particular enhanced image slice.

In order to automatically extract the heart region from the rest of the image slice, we propose a morphology-based masking scheme. This includes morphological “opening by reconstruction” operation, followed by thresholding and subsequent morphological dilation operator. We use circular (disk-shaped) structuring element (SE) for morphological operators due to mainly circular shape of the heart (in 2D image slice), its chambers, and proximal vessels. We determine the optimal size of SEs based on the predicted size of the heart (input provided by cardiologists) and pixel spacing (contained in the DICOM header). In our case, the calculated SE size of the morphological operators is 40 pixels and 20 pixels for opening by reconstruction and dilation operator, respectively. The intermediate thresholding depends on each input image slice statistics and is therefore applied differently to each input image slice. The result of automatic ROI extraction is shown in Figure 3 for the image slice in Figure 2, showing the accurate delineation of the whole heart region.

Alternatively, the extracted ROI can be shown as a mask (binary) image *X*, where all pixels corresponding to nonheart region are assigned gray value 0, while the heart pixels are assigned gray value 1:

(1) $$\begin{array}{c} X\left(i,j\right)=\left\{\begin{array}{ll} 1, & if\;pixel\;\left(i,j\right)\;belongs\;to\;the\;region\;of\;interest,\\ 0, & otherwise. \end{array}\right. \end{array}$$

### 2.3. Epicardial Fat Segmentation Scheme

In this section, we describe the slice-by-slice epicardial fat segmentation scheme featuring high level of adaptivity, accuracy, and automatization. In the proposed segmentation approach, we first perform image clustering on previously preprocessed input image within the extracted ROI as shown in Figure 3. This represents an initial segmentation corresponding to a single cluster, which is then further processed to only extract parts corresponding to the epicardial fat tissue. The main steps in the proposed scheme are fuzzy c-means clustering approach, ellipse fitting, and final subclustering/filtering of the ellipse-shaped cluster region corresponding to the epicardial fat only.

#### 2.3.1. Fuzzy c-Means Clustering in the Region of Interest

The proposed initial segmentation of the preprocessed input image slice (within the ROI) is performed using fuzzy c-means clustering [22] with predefined number of clusters *C*. The feature vector used for clustering in the segmentation task is *u* = [*u*_1_*u*_2_]^*T*^, where *u*_1_ represents the mean value of luminance of the particular *R* × *R* image pixel patch *U* = {*U*(*i*, *j*) | *i*, *j* = 1,…, *R*}, while *u*_2_ represents the variance of the luminance in that patch:

(2) $$\begin{array}{c} u_{1}=\frac{1}{R^{2}}\overset{R}{\underset{i,j=1}{\sum }}U\left(i,j\right),\\ u_{2}=\frac{1}{R^{2}}\overset{R}{\underset{i,j=1}{\sum }}{\left(U\left(i,j\right)-u_{1}\right)}^{2}. \end{array}$$

Patches within the ROI are sampled and gathered for each image slice. Features are calculated so that we obtain a set *𝒮* = {*u*_*k*_ | *k* = 1,…, *Q*} of features on which we perform clustering. Note that *Q* is the number of uniformly sampled patches from the image slice and that we have a unique correspondence between the index of features *k* in *𝒮* and centers (coordinates) of patches *p*_*k*_ = [*i*^(*k*)^, *j*^(*k*)^]^*T*^, where *k* = 1,…, *Q*. The number of clusters *C* is predefined and is determined empirically depending of the particular CT scanning aperture. The clustering is performed without initial seed information and generates *𝒢* = {*μ*_*c*_(*k*) | *k* = 1,…, *Q*, *c* = 1,…, *C*} fuzzy clusters according to their soft characteristic functions and satisfying *μ*_*c*_(*k*) ∈ [0, 1], for *c* = 1,…, *C* and *k* = 1,…, *Q*. Note that the closer *μ*_*c*_(*k*) is to 1 indicates a higher correspondence of a particular center of the patch *p*_*k*_ the cluster *c*.

In order to obtain reference image patch, user is required to inspect single representative CT slice (from the target patient data) and place a seed point. The seed point represents the image coordinates of the center (*i*^∗^, *j*^∗^) of the reference patch *U*^∗^. The proposed unsupervised clustering approach determines the resulting cluster, that is, selects the cluster that corresponds to the epicardial fat. We obtain that by calculating the distance between the reference patch *U*^∗^ and all *C* fuzzy clusters contained in *𝒢*. For the fixed fuzzy cluster with index *c*, we determine the distance as follows:

(3) $$\begin{array}{c} d\left(c,U^{*}\right)={\left\|u^{*}-{\hat{u}}_{c}\right\|}_{2}, \end{array}$$

where ${\hat{u}}_{c}$ is the mean of the feature vectors from *𝒮*, pondered by corresponding soft characteristic function *μ*_*c*_(*k*), that is,

(4) $$\begin{array}{c} {\hat{u}}_{c}=\frac{1}{Q}\overset{Q}{\underset{k=1}{\sum }}\mu_{c}\left(k\right)u_{k}. \end{array}$$

We obtain the resulting cluster, as the cluster with minimal distance to the reference patch *U*^∗^, that is, the cluster with index *c*^res^ given by

(5) $$\begin{array}{c} c^{res}={\arg\min}_{c}d\left(c,U^{*}\right). \end{array}$$

Note that each pixel with spatial coordinate (*i*, *j*), *i* = 1,…, *M* and *j* = 1,…, *N* (image slices are of *M* × *N* size), can be covered by multiple patches (unless patch sampling is disjoint). Since we need the resulting fuzzy cluster on a discrete set of pixels, the resulting pixel-wise fuzzy cluster *c*^res^ is obtained using the following equation:

(6) $$\begin{array}{c} \mu_{c^{res}}\left(i,j\right)=\frac{1}{\left|H_{i,j}\right|}\sum_{h\in H_{i.j}}\mu_{c^{res}}\left(k\left(h\right)\right), \end{array}$$

where *H*_*i*,*j*_ is the set of all patches that cover pixel (*i*, *j*) and *k*(*h*) is the index of *h*th patch within the fuzzy cluster set in *𝒢*. The resulting fuzzy cluster with index *c*^res^, recalculated using (6) and defined on the set of particular image slice pixels, is the output cluster from the clustering step. The resulting cluster contains pixels corresponding to epicardial fat tissue, and its cluster data is used in further processing.

**Figure 4:**
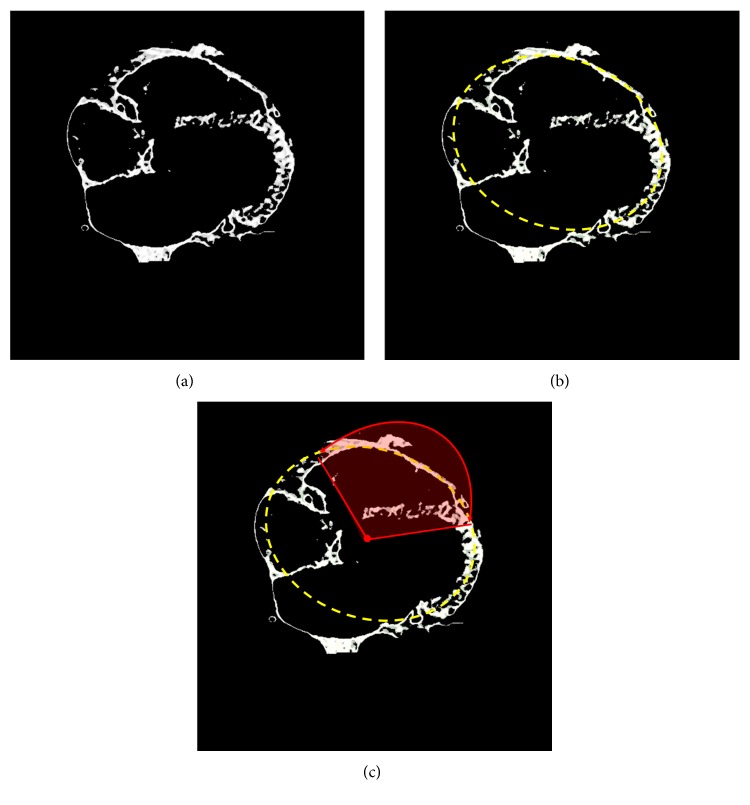
The resulting fuzzy cluster that is considered to contain epicardial fat tissue, for the image slice example shown in Figure 3. White color-marked regions show clustered data (a), yellow color-marked pixels indicate ellipsoidal shape fitting approach (b), and red color-marked lines denote the angular bandwidth constrain (c).

In Figure 4(a), we show the resulting fuzzy cluster for the input image slice shown in Figure 3. The resulting cluster contains epicardial fat tissue with other outlier data that we need to remove further.

#### 2.3.2. Cluster Data Postfiltering Using Geometric Ellipse Fitting

After completing the fuzzy c-mean-based initial segmentation (as explained in Section 2.3.1), we perform postfiltering of the extracted cluster data using a geometric ellipse fitting. This step is used for filtering out undesired parts of the target cluster data.

As shown in Figure 4(b), the upper parts of the cluster data can be well fitted with an ellipse denoted with yellow dashed lines. As confirmed by physicians, the epicardial fat distribution corresponds to the fitted ellipse in Figure 4. Hence, our assumption is that significant parts of the resulting cluster, which the medical doctors would classify as epicardial fat tissue, are mainly located around the fitted ellipse. In this assumption, we denote *Geometric Ellipse Prior* (GEP). The ellipse, denoted as *Referent Ellipse* (RE), is placed at the center of the cluster and passing through the bordering parts, as depicted in Figure 4(b). As we expect the epicardial fat tissue to be placed around the ventricles, mainly anterolateral surface, we incorporate the mentioned assumption in the additional geometric prior, also related to the GEP. In addition to the geometric ellipse fitting, we apply constrain on the angle that parametrizes RE so that parts of the resulting cluster within the predefined arc of the RE are selected as desirable and the other parts are filtered out as undesirable. The latter is shown in Figure 4(c) with red colored arc.

Computation of the GEP and corresponding RE for the particular image slice is done in the following three steps:

1. Estimation of the RE center (*x*_*c*_, *y*_*c*_)
2. Estimation of the angle *φ*_0_ between the major axes of the RE and the *x* referent axes of image slice
3. Estimation of the RE parameters *a* and *b* corresponding to two characteristic axes that define the shape of the ellipse.

**Figure 5:**
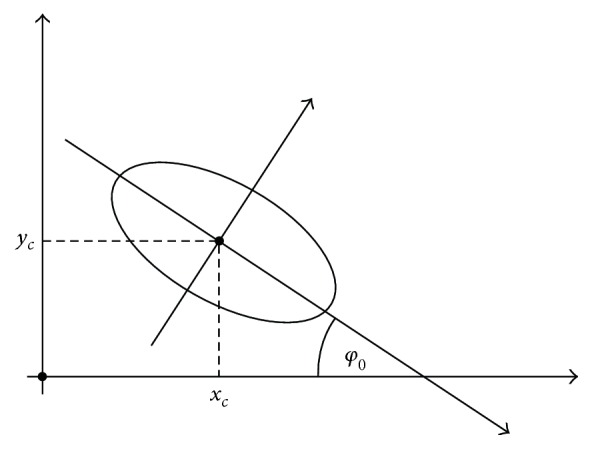
Figure depicting ellipse which parameters need to be estimated.

The RE is parameterized and represented in polar coordinates and given in the following form:

(7) $$\begin{array}{c} \begin{array}{l} x\left(\varphi \right)=x_{c}+a\cos\left(\varphi +\varphi_{0}\right),\\ y\left(\varphi \right)=y_{c}+b\sin\left(\varphi +\varphi_{0}\right),\\ \varphi \in \left[0,2\pi \right), \end{array} \end{array}$$

where (*x*_*c*_, *y*_*c*_) ∈ ℝ^2^ is the center of an ellipse, *a*, *b* > 0 are parameters corresponding to characteristic axes of an ellipse, and *φ* ∈ [0, 2*π*) is an angular parameter (see Figure 5). For estimating (*x*_*c*_, *y*_*c*_), we use *the center of gravity* approach, where the estimates (*x*_*c*_, *y*_*c*_) are determined as

(8) $$\begin{array}{c} {\hat{x}}_{c}=\frac{M_{\mu_{c^{res}}I}\left(1,0\right)}{M_{\mu_{c^{res}}I}\left(0,0\right)},\\ {\hat{y}}_{c}=\frac{M_{\mu_{c^{res}}I}\left(0,1\right)}{M_{\mu_{c^{res}}I}\left(0,0\right)}, \end{array}$$

with moments *M*_*μ*_*c*^res^_*I*_(*p*, *q*), *p*, *q* ∈ N, defined by

(9) $$\begin{array}{c} M_{\mu_{c^{res}}}\left(p,q\right)=\overset{M}{\underset{i=1}{\sum }}\overset{N}{\underset{j=1}{\sum }}i^{p}j^{q}X\left(i,j\right)\mu_{c^{res}}\left(i,j\right), \end{array}$$

where *i* and *j* are discrete spatial coordinates corresponding to *x*- and *y*-axes of the image slice, that is, referent coordinate system in which the RE is supposed be positioned. Further on, in (8), *X* stands for the pixel luminance that is weighted by the characteristic function of the resulting fuzzy cluster *μ*_*c*^res^_. In such manner, we do not only take into account pixel luminance value but also the membership degree of the particular pixel to the resulting fuzzy cluster (if we consider pixels to be statistical observations with mass density function *μ*_*c*^res^_, *M*(1, 0) = *E*_*i*_(·), and *M*(0, 1) = *E*_*j*_(·), the previous expression can be considered as mathematical expectation for given discrete spatial coordinates *i* and *j*, resp.).

Next, we need to estimate the angle *φ*_0_ between the major axes of the RE and the *x* referent axes of the image slice (see Figure 5), which defines the main direction of the “soft point cloud” in the resulting cluster as well as orientation of the RE. Therefore, we define the vector of observations *𝒫* as {*p*_*i*_ = *μ*_*c*^res^_(*i*, *j*)[*i*, *j*]^*T*^ | (*i*, *j*) belongs to ROI} and estimate the covariance matrix *C*_*P*,*P*_ = *E*((*P* − *E*(*P*))(*P* − *E*(*P*))^*T*^), as

(10) $$\begin{array}{c} {\hat{C}}_{P,P}=\frac{1}{\left|P\right|}\overset{\left|P\right|}{\underset{i=1}{\sum }}\left(\left(p_{i}-\hat{P}\right){\left(p_{i}-\hat{P}\right)}^{T}\right),\\ \hat{P}=\overset{\left|P\right|}{\underset{i=1}{\sum }}p_{i}, \end{array}$$

where we use *μ*_*c*^res^_ in order to incorporate additional information regarding the soft characteristic function for the resulting cluster *c*^res^ (5). For the purpose of determining *φ*_0_, we apply principle component analyses (PCA): we decompose the positive definite matrix ${\hat{C}}_{P,P}^{\varepsilon }={\hat{C}}_{P,P}+\varepsilon I$ which is regularized by diagonal loading of ${\hat{C}}_{P,P}$ with small positive *ε* > 0. As a result, we obtain the following: ${\hat{C}}_{P,P}^{\varepsilon }=V\Lambda V^{T}.$ Here, *Λ* = diag(*λ*_1_, *λ*_2_) and *V* = [*v*_1_ | *v*_2_], where *λ*_*i*_ > 0 and *v*_*i*_ ∈ R^2^, *i* = 1, 2 are eigenvalues and eigenvectors of the matrix ${\hat{C}}_{P,P}^{\varepsilon },$ respectively. Additionally, *V* is unitary matrix, where *v*_1_⊥*v*_2_, and ‖*v*_*i*_‖ = 1, *i* = 1, 2.

The main direction of the “soft point cloud,” representing the resulting cluster, is actually the direction of eigenvector *v*^∗^ corresponding to the largest eigenvalue, namely, $v^{*}=v_{\hat{i}}$, such that $\hat{i}=\text{arg }{\text{max}}_{i}\left\{\lambda_{i}\right\}.$ Finally, we obtain the estimate of *φ*_0_ as

(11) $$\begin{array}{c} {\hat{\varphi }}_{0}=\arccos\left(\mathbb{P}{roj}_{e_{x}}\left(v^{*}\right)\right), \end{array}$$

where Proj_*e*_*x*__(*v*^∗^) = (*v*^∗^)^*T*^*e*_*x*_ is the projection of *v*^∗^ to the unit *x*-axis vector *e*_*x*_. We approximate the projection by simplified affine transformation applied to the data points belonging to the ROI:

(12) $$\begin{array}{c} \left[\begin{array}{c} \tilde{x}\\ \tilde{y} \end{array}\right]=R\left(-{\hat{\varphi }}_{0}\right)\left[\begin{array}{c} x\\ y \end{array}\right]-\left[\begin{array}{c} {\hat{x}}_{c}\\ {\hat{y}}_{c} \end{array}\right], \end{array}$$

with

(13) $$\begin{array}{c} R\left(-{\hat{\varphi }}_{0}\right)=\left[\begin{array}{cc} \cos{\hat{\varphi }}_{0} & \sin{\hat{\varphi }}_{0}\\ -\sin{\hat{\varphi }}_{0} & \cos{\hat{\varphi }}_{0} \end{array}\right] \end{array}$$

being rotation matrix for angle $-{\hat{\varphi }}_{0}.$ Such projection matrix is applied on each (*x*, *y*) position in the image slice. Further on, we also apply (12) to the original RE defined by (7), which will then be centered in (0, 0) with *x*-axis aligned with the principle axes (${\hat{\varphi }}_{0}=0$). The new transformed RE represented by the following parametric equation:

(14) $$\begin{array}{c} x_{t}\left(\varphi \right)=a\cos\varphi ,\\ y_{t}\left(\varphi \right)=b\sin\varphi ,\\ \varphi \in \left[0,2\pi \right). \end{array}$$

Finally, in order to estimate the *a*, *b* > 0 parameters, which define the two characteristic axes of the RE, we aim at solving the following constrained optimization problem:

(15) $$\begin{array}{c} \begin{array}{c} \left\{\hat{a},\hat{b}\right\}={\arg\min}_{a,b}\overset{M}{\underset{i=1}{\sum }}\overset{N}{\underset{j=1}{\sum }}X\left(i,j\right)\mu_{c}^{res}\left(i,j\right)\left[{\left(x_{t}\left(\varphi_{i,j}\right)-\tilde{x}\left(i,j\right)\right)}^{2}+{\left(y_{t}\left(\varphi_{i,j}\right)-\tilde{y}\left(i,j\right)\right)}^{2}\right]\\ s.t.\;0<B_{l}<a,b<B_{u}, \end{array} \end{array}$$

where *x*_*t*_(*φ*) and *y*_*t*_(*φ*) are given in parametric form by (14), so that (*x*_*t*_, *y*_*t*_) belongs to the transformed RE (whose parameters *a* and *b* we actually estimate) and $\tilde{x}$ and $\tilde{y}$ correspond to transformed coordinates (by (12)) of the image slice data samples. Additionally, $\varphi_{i,j}=\text{arctan}\left(\tilde{y}\left(i,j\right)/\tilde{x}\left(i,j\right)\right),$*i* = 1,…, *M*, *j* = 1,…, *N*, while terms *B*_*l*_, *B*_*u*_ > 0 bound the admissible set parameters from above, in order to make search space smaller. Since the optimization problem (15) is not convex, we solved it by finding the local minimum using standard gradient procedures.

**Figure 6:**
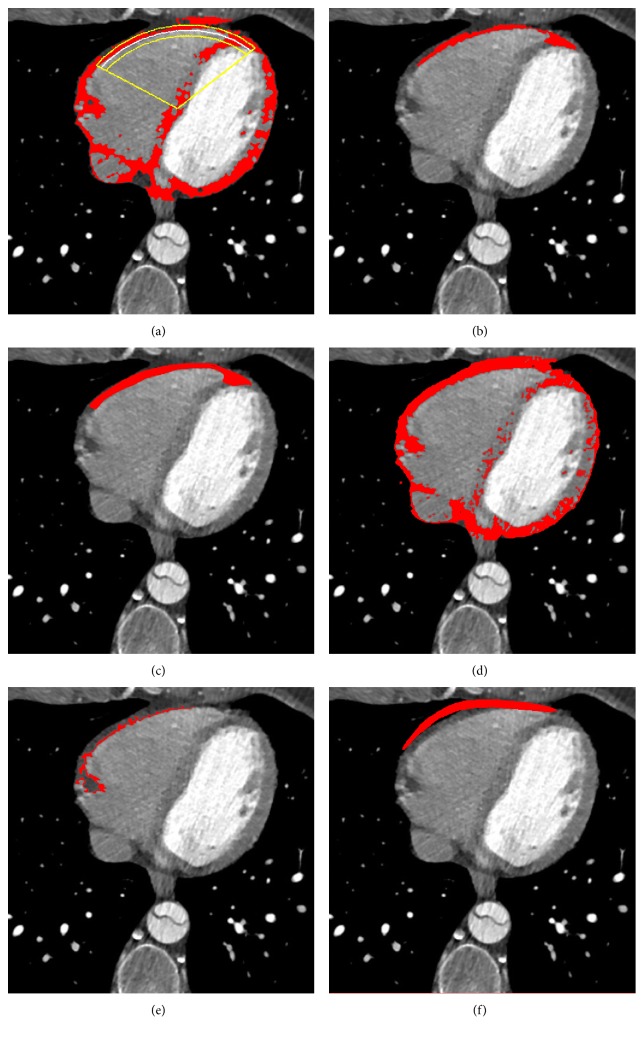
Results for one particular CT image slice by the proposed segmentation method in comparison to the manual segmentation obtained by medical doctors and the three reference methods (snake, region growing, and LAMT): (a) *α*-cut of the resulting cluster; (b) the proposed segmentation approach; (c) manual segmentation performed by medical doctors; (d) LAMT [18]; (e) region growing [21]; (f) snake [20].

After we obtain the optimal parameters for the RE, we aim at filtering out the nonepicardial fat parts of the resulting cluster. According to the GEP assumption, these are the segments of the resulting fuzzy cluster that are out of the *ε* neighborhood of the RE. Parameter *ε* > 0 has been found empirically and has been fixed for all slices and all patients in all experiments. In addition to the obtained RE and *ε* neighborhood ring, we introduce two arcs to constrain the part of the RE within which the resulting cluster will be marked as desirable (to be retained), while the rest will be undesirable (to be removed), as shown in Figure 6. The angle between the two arcs is defined as *φ*_*C*_ = *φ*_2_ − *φ*_1_, *φ*_1_ < *φ*_2_, where *φ*_1_ and *φ*_2_ represent the first and second arc, starting from *x*-axis, respectively.

In order to determine the *φ*_1_ and *φ*_2_, that is, the desirable area, we first perform the defuzzification of the resulting cluster. We compute defuzzified crisp cluster *ℳ* = (*μ*_*c*^res^_)_*α*_ by *α*-cutting the resulting fuzzy cluster *μ*_*c*^res^_ with predefined *α* ∈ (0, 1). (The parameter *α* has been found empirically, and it has been fixed for all slices and all patients in all experiments.) As a result, we have (*μ*_*c*^res^_)_*α*_ = {(*i*, *j*) | *μ*_*c*^res^_(*i*, *j*) ≥ *α*} based on which we obtain the desirable parts *ℳ*^∗^ of the resulting fuzzy cluster *μ*_*c*^res^_, that is, crisp cluster *ℳ*, in which pixels satisfy the following:

(16) $$\begin{array}{c} M^{*}=\left\{\left.\left(i,j\right)\in M\right|\varphi_{i,j}\in \left[\varphi_{1},\varphi_{2}\right],d\left(\left(i,j\right),RE\right)\leq \varepsilon \right\}, \end{array}$$

where $\varphi_{i,j}=\text{arctan}\left(\tilde{y}\left(i,j\right)/\tilde{x}\left(i,j\right)\right)$ represents the angular polar coordinate corresponding to particular pixel (*i*, *j*) ∈ *ℳ* and *d*((*i*, *j*), RE) represents the distance between the pixel (*i*, *j*) ∈ *ℳ* and the RE:

(17) $$\begin{array}{c} d\left(\left(i,j\right),RE\right)=\left|{\tilde{\rho }}_{i,j}-\rho_{RE}\left(\varphi_{i,j}\right)\right|, \end{array}$$

where

(18) $$\begin{array}{c} {\tilde{\rho }}_{i,j}=\sqrt{{\tilde{x}}^{2}\left(i,j\right)\cos^{2}\varphi_{i,j}+{\tilde{y}}^{2}\left(i,j\right)\sin^{2}\varphi_{i,j}} \end{array}$$

represents the radius polar coordinate corresponding to (*i*, *j*) ∈ *ℳ* and

(19) $$\begin{array}{c} \rho_{RE}\left(\varphi_{i,j}\right)=\sqrt{{\hat{a}}^{2}\cos^{2}\varphi_{i,j}+{\hat{b}}^{2}\sin^{2}\varphi_{i,j}} \end{array}$$

represents the radius polar coordinate corresponding to point on the RE, with the angular polar coordinate equal to *φ*_*i*,*j*_. ($\hat{a}$ and $\hat{b}$ are defined in (15).)

The *ℳ*^∗^ actually represents the final 2D segmentation of epicardial fat for particular CT image slice. In our experiments, the angles *φ*_1_ and *φ*_2_ have been determined empirically for particular type of patients and CT imaging parameters. For this purpose, we have used assistance of physicians to set *the control angles* on training set of patients. The control angles are represented by *φ*_1_^*l*^ and *φ*_2_^*l*^, *l* = 1,…, *L*, which are manually set for reduced number of equidistant control image slices. The actual *φ*_1_ and *φ*_2_ for each CT slice are then determined as a linear interpolation of the existing neighboring control angles. The final *φ*_1_ and *φ*_2_ (for all CT slices) are then used in test phase for segmentation of the data corresponding to same type of patients and CT imaging parameters. As the final postprocessing step, we perform the morphological image closure operation (with the circle-shaped structuring element) of the region *ℳ*^∗^, thus yielding the final result of epicardial fat segmentation on a particular CT image slice.

## 3. Results and Discussion

We present in this section the results of the proposed semiautomatic epicardial fat segmentation in terms of objective segmentation measure and clinical evaluation. The manual segmentation performed by physicians is used as ground truth data for computation of objective 3D segmentation measures. The “leave one out” cross-validation principle was used in training and testing phase. We compare the segmentation results of the proposed approach to the results obtained by our previous method for epicardial fat segmentation [18] (which we call local adaptive morphology-thresholding (LAMT), snake active contour method [20], and region growing method [21]). (The implementation of the snake and region growing algorithms has been used from open-source Matlab code freely available on http://www.mathworks.com.)

We seek the optimal values for the following parameters: *ms*1, *ms*2, *α*, and *ε*. Parameter (diameter in the number of pixels) *ms*1 is the size of the disk-shaped structural element for the morphological operation “opening by reconstruction,” used in the preprocessing phase of extracting the heart region (region of interest) from the rest of the image slice. Parameter *ms*2 is the size of the disk-shaped structural element for the morphological operation “dilation,” which follows the “opening by reconstruction” operation, both performed on contrast-enhanced input image, as explained in Section 2.2.

The parameter *α* ∈ (0, 1) is used for obtaining the *α*-cut in the defuzzification phase, as the part of obtaining the final segmentation result for the particular image slice, which is described in Section 2.3.2. Parameter *ε*, given in the number of pixels, defines the neighborhood of geometric ellipse used in postfiltering phase, described in Section 2.3.2. In order to obtain optimal values for the mentioned parameters, we form the following discrete combination set: *ms*1 ∈ {30, 35, 40, 45, 50, 55, 60}, *ms*2 ∈ {5, 10, 15, 20, 25, 30}, *α* ∈ {0.4, 0.5, 0.6, 0.7, 0.8}, and *ε* ∈ {5, 10, 15, 20, 25, 30}. On the previously described combination set (passing all possible values), and along the lines of “leave one out” cross-validation training/testing procedure, we evaluate the cost function. The cost function, for this purpose, is defined as the average of the Dice similarity coefficients between the processed and the manually segmented (by the medical expert) ground truth image slices belonging to the training set of patient. As we use “leave one out” cross-validation procedure, in each round of the procedure, we randomly chose, by uniform distribution, one patient for testing and the rest to be in the training set. The described cost is then evaluated on all CT image slices belonging to the training set. We obtained “the best” values of those parameters as (*ms*1, *ms*2, *α*, *ε*) = (40, 20, 0.7, 20).

The number *L* of equidistant control CT image slices for obtaining the control bordering angles *φ*_1_ and *φ*_2_ is chosen to be large enough, but still small enough as well, not to be too demanding and time consuming for the medical expert. The medical experts had to label those *L* slices for each patient, and as a result, the bordering control angles *φ*_1_ and *φ*_2_ were obtained, along with the previously mentioned (*ms*1, *ms*2, *α*, *ε*) within the “leave one out” cross-validation training/testing procedure. Namely, in each round of the cross-validation procedure, for each *l* = 1,…, *L*, we obtain the resulting bordering control angles *φ*_1_^(*l*)^ and *φ*_2_^(*l*)^, by simple averaging over all patients chosen to be in the training set in that particular round. Then, the testing is performed, for that particular round. The final control angles are chosen to be those obtaining the lowest cost (the average of the Dice similarity coefficients between the processed and the manually segmented slices) of all rounds. As stated in Section 2.3.2, for all other patients and CT slices not belonging to the resulting bordering control slices, the bordering angles *φ*_1_ and *φ*_2_ are determined by interpolation of the existing neighboring control angles. Finally, the lower and upper bounds *B*_*l*_ and *B*_*u*_, used in (15), are obtained empirically and set to *B*_*l*_ = 200 and *B*_*u*_ = 450 in all our experiments.

**Figure 7:**
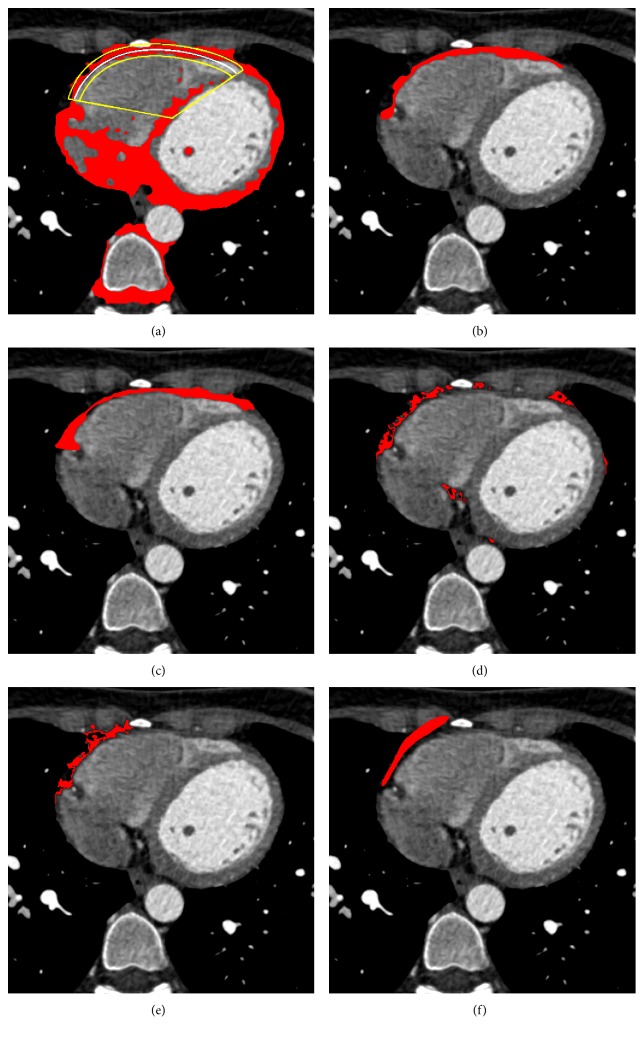
Results for one particular CT image slice by the proposed segmentation method in comparison to the manual segmentation obtained by medical doctors and the three reference methods (snake, region growing, and LAMT): (a) *α*-cut of the resulting cluster; (b) the proposed segmentation approach; (c) manual segmentation performed by medical doctors; (d) LAMT [18]; (e) region growing [21]; (f) snake [20].

**Figure 8:**
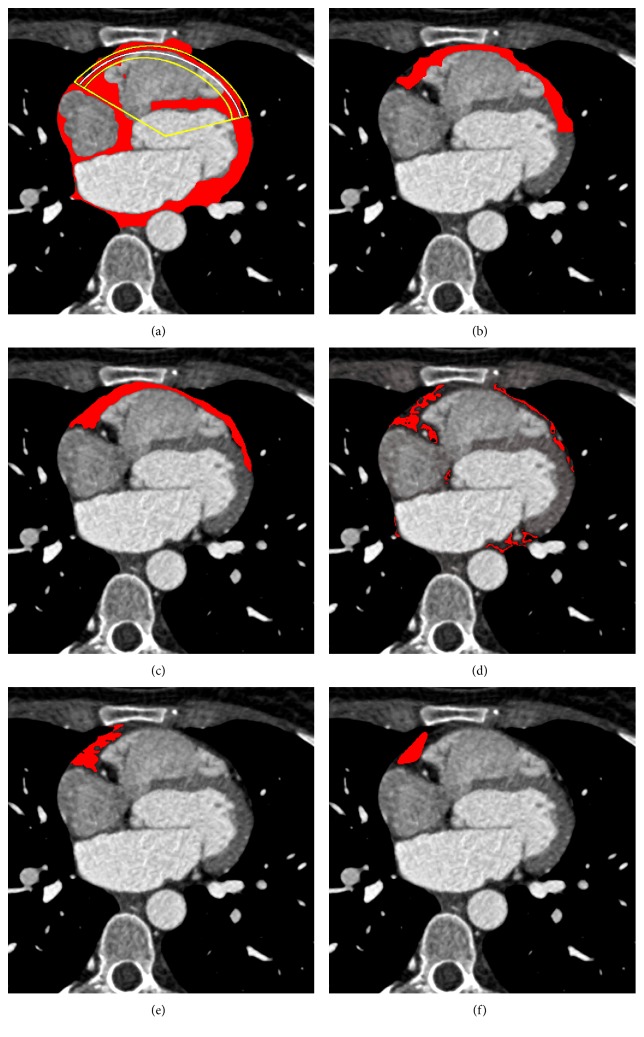
Results for one particular CT image slice by the proposed segmentation method in comparison to the manual segmentation obtained by medical doctors and the three reference methods (snake, region growing, and LAMT): (a) *α*-cut of the resulting cluster; (b) the proposed segmentation approach; (c) manual segmentation performed by medical doctors; (d) LAMT [18]; (e) region growing [21]; (f) snake [20].

In Figures 6(a), 7(a), and 8(a), we show, for three different examples of the input CT slices, the *α*-cut of the resulting fuzzy cluster along with the *ε*, *φ*_1_, and *φ*_2_ neighborhood of RE corresponding to the desirable area, with applied postprocessing step of morphological operation. In Figures 6(a), 7(a), and 8(a), red color-marked region denotes resulting fuzzy cluster that is subsequently fitted by the proposed RE. Additionally, the proposed *α*-cut RE is shown in the same figures with yellow and white lines, where white line corresponds to the *α*-cut part of the estimated RE and yellow lines indicate *ε* neighborhood and angular bandwidth of the selected part.

The segmentation results for a single CT slices are further shown in Figures 6, 7, and 8, where the proposed segmentation approach is compared to manual segmentation (done by medical doctors) and the reference three baseline methods. Red color-marked regions in the figures denote resulting segmented epicardial fat regions, obtained by applying particular method. It can be observed that the proposed method outperforms the three baseline methods ([18, 20, 21]), in terms of lower number of erroneously detected regions and higher degree of connectivity of the detected region, relatively to the reference (manually segmented ground truth).

In order to compare the segmentation results of our method to the ground truth, we use Dice similarity coefficient [23], which is the set similarity measure defined for two sets *A* and *B* (in our case, set of voxels belonging to segmented images and the ground truth image) as *D*(*A*, *B*) = (2|*A*∩*B*|)/(|*A*| + |*B*|), where |·| of voxels within the particular set.

In Table 1, we show the results for the computed Dice coefficients, by which we measure the difference between automatic and manually obtained segmentation (approved by the medical expert) for the whole 3D image/volume, for the proposed and three reference segmentation methods, namely, snake active contour method [20], region growing method [21], and the LAMT method [18]. It can be observed that the results of our proposed method yield high Dice coefficient values and that are significantly higher than the Dice coefficients obtained for the reference methods that we compared to. We also note that the proposed method is less time consuming for the medical expert in comparison to the reference methods. Namely, snake active contour method requires initial contour to be delivered for every CT image slice; region growing method demands coordinates of at least one patch to be delivered for each CT slice; and LAMT method requires coordinates of four patches to be provided.

In order to draw clinical conclusions from the measured epicardial fat volumes, we introduce *Normalized Volume* (NV) of epicardial fat as a ratio of the epicardial fat volume and weight of the patient. The calculated NV values are given in Table 2 together with relative error of epicardial fat measured by comparing the segmented (by the proposed method) and manually annotated volume. The results of relative error calculation show acceptable error rates (less or equal to 15%) with two outliers.

Our experiments indicate higher NV values for 3-vessel coronary artery disease than in case of 2-vessel coronary artery disease with average values 0.96 and 0.75, respectively. Also, patients with hypertension have exhibited slightly lower NV values than patients with normal blood pressure, with average values 0.82 and 0.96, respectively. Out of all test subjects, 3 patients had reduced ejection fraction (bellow 42%). However, the reduced ejection fraction does not correlate well enough with calculated NV fat volume.

## 4. Conclusion

Segmentation and quantification of epicardial fat in cardiac CT images play an important role in assisting physicians in diagnosing cardiovascular diseases and progressing their outcomes. The higher level of automation of this process helps to reduce interobserver variability and shorten execution times. In this work, a novel epicardial fat segmentation scheme is proposed. The method is based on local adaptive morphology and fuzzy c-means clustering, where the only user input is the coordinates of the particular image patch belonging to the target epicardial fat area. Additionally, geometric ellipse prior is used in order to filter out the undesired parts of the target cluster. Experimental results presented demonstrate the good correspondence between the proposed method and the manual ground truth segmentation.

## Figures and Tables

**Table 1:** Comparison of methods using Dice similarity coefficient with manually segmented ground truth images (approved by a cardiovascular surgeon). Our proposed method outperforms the LAMT method, the classical region growing, and the snake methods.

Patient	1	2	3	4	5	6	7	8
Proposed method	0.76	0.65	0.6	0.7	0.73	0.54	0.73	0.82
LAMT	0.3	0.32	0.38	0.24	0.5	0.2	0.3	0.07
Region growing	0.39	0.15	0.34	0.34	0.28	0.24	0.39	0.17
Snake	0.3	0.15	0.16	0.32	0.36	0.29	0.38	0.3

**Table 2:** Volume measurement error between our proposed method and manually segmented ground truth images.

Patient	1	2	3	4	5	6	7	8
Normalized volume (voxels)	1.1	0.73	0.43	0.68	0.96	0.95	1.38	0.91
Volume relative error (%)	1.5	15	14.5	29.6	33.8	8.7	14.6	9.5
Coronary artery disease (vessels)	3	2	3	2	3	2	3	3
Hypertension ejection	No	Yes	No	Yes	No	Yes	No	Yes
Fraction (vessels)	60	37	26	63	42	55	56	60
